# GAT: A Graph-Theoretical Analysis Toolbox for Analyzing Between-Group Differences in Large-Scale Structural and Functional Brain Networks

**DOI:** 10.1371/journal.pone.0040709

**Published:** 2012-07-13

**Authors:** S. M. Hadi Hosseini, Fumiko Hoeft, Shelli R. Kesler

**Affiliations:** 1 Department of Psychiatry and Behavioral Sciences, Stanford University School of Medicine, Stanford, California, United States of America; 2 Division of Child and Adolescent Psychiatry, Department of Psychiatry, University of California San Francisco, San Francisco, California, United States of America; 3 Stanford Cancer Center, Palo Alto, California, United States of America; University of Namur, Belgium

## Abstract

In recent years, graph theoretical analyses of neuroimaging data have increased our understanding of the organization of large-scale structural and functional brain networks. However, tools for pipeline application of graph theory for analyzing topology of brain networks is still lacking. In this report, we describe the development of a graph-analysis toolbox (GAT) that facilitates analysis and comparison of structural and functional network brain networks. GAT provides a graphical user interface (GUI) that facilitates construction and analysis of brain networks, comparison of regional and global topological properties between networks, analysis of network hub and modules, and analysis of resilience of the networks to random failure and targeted attacks. Area under a curve (AUC) and functional data analyses (FDA), in conjunction with permutation testing, is employed for testing the differences in network topologies; analyses that are less sensitive to the thresholding process. We demonstrated the capabilities of GAT by investigating the differences in the organization of regional gray-matter correlation networks in survivors of acute lymphoblastic leukemia (ALL) and healthy matched Controls (CON). The results revealed an alteration in small-world characteristics of the brain networks in the ALL survivors; an observation that confirm our hypothesis suggesting widespread neurobiological injury in ALL survivors. Along with demonstration of the capabilities of the GAT, this is the first report of altered large-scale structural brain networks in ALL survivors.

## Introduction

Brain structural and functional connectivity plays an important role in neuroanatomy, neurodevelopment, electrophysiology, functional brain imaging, and neural basis of cognition [1]. Brain networks, along with other biological networks, have been shown to follow a specific topology known as small-world. A small-world network architecture facilitates rapid synchronization and efficient information transfer with minimal wiring cost through an optimal balance between local processing and global interaction [2]. Since small-world characteristics were described quantitatively for brain networks, there have been multiple graph-theoretical studies seeking to assess the organization of structural and functional brain networks in healthy individuals and patient population [3]–[22].

The unique feature of graph-theoretical analysis, compared with the more traditional univariate neuroimaging approaches, is that it can directly test the differences in topological parameters of the brain network such as small-worldness, modularity, highly connected regions (hubs), and regional network parameters. [23], [24] Additionally, graph theoretical analysis is potentially applicable to any modality, scale, or volume of neuroscientific data [25]. Graph theoretical analyses have been applied to regional gray matter volume, cortical thickness, surface area, and diffusion weighted imaging data to analyze topology of structural brain networks and to resting state and task-related functional connectivity data to analyze the topology of functional brain networks. These studies have illustrated an alteration of arrangements in structural and functional brain networks associated with normal aging, multiple sclerosis, Alzheimer’s disease, schizophrenia, depression, and epilepsy [4], [5], [9], [12], [14], [15], [20], [22], [26].

In recent years, a number of freely available software packages have been introduced to apply graph theory for analyzing topology of brain networks (e.g. Brain Connectivity Toolbox [27]; eConnectome [28]; NetworkX (http://networkx.lanl.gov/overview.html); and Brainwaiver (http://cran.r-project.org/web/packages/brainwaver). The focus of these packages is mainly on extracting network measures and/or visualization of networks. However, the methodology of comparing network topologies of different groups (or systems) is challenging [29]. In this report, we describe the development a graph analysis toolbox (GAT) that facilitates analysis and comparison of structural and functional brain networks. GAT is an open-source Matlab-based package with graphical user interface that integrates the Brain Connectivity Toolbox [27] for quantification of network measures and the REX toolbox (http://web.mit.edu/swg/software.htm) for region of interest extraction (REX). For structural network analysis, GAT accepts gray matter volume/surface area/cortical thickness data of groups, extracts structural correlation networks, applies different thresholding schemes for comparing networks between groups, calculates network measures for different thresholding schemes, estimates between-group differences in network measures using functional data analysis (FDA) [30], [31] and area under the curve (AUC) analysis, tests the significance of between-group differences in global and regional network measures using nonparametric permutation testing, and performs hub analysis, random failure and targeted attack analysis and modularity analysis. For functional networks, GAT accepts the output from functional connectivity toolbox (http://www.nitrc.org/projects/conn), extracts the network measures, finds the range of network densities where individual networks are not fragmented, performs both parametric and non-parametric statistical tests to test the significance of between-group differences in global and regional network measures at each densities as well as on FDA and AUC estimates, and the above-mentioned analyses as for structural graphs.

To demonstrate the capabilities of GAT, we investigated the differences in organization of structural brain networks in survivors of acute lymphoblastic leukemia (ALL), the most common childhood cancer, and healthy matched controls. There are several lines of evidence suggesting that ALL may involve widespread neurobiologic injury. First, while the mechanism by which cancer and its treatments affect cognitive function are largely unknown, possible candidates include neurotoxic effects of chemotherapy, oxidative damage and cytokine dysregulation [32], [33]. These candidate mechanisms might have diffuse effects on brain structure. Second, structural neuroimaging studies, including our own [34] have shown diffuse changes in white matter and gray matter structure associated with ALL [35]–[38]. Third, meta-analyses of neuropsychological studies on ALL survivors have indicated decline in a wide range of cognitive functions including executive functioning, processing speed and memory [39], [40] (see [41], [42] for a review). These functions are known to be subserved by distributed, integrated neural networks [43]. We investigated whether topological properties of large-scale structural brain networks are altered in ALL survivors.

## Materials and Methods

### Overview of How to Use the GAT

**Figure 1:**
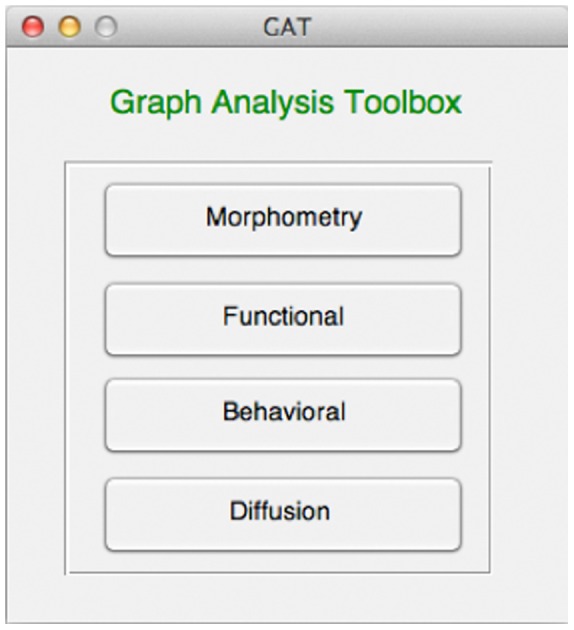
Main GUI of the graph analysis toolbox (GAT). The toolbox allows topological assessment of morphometry, functional, diffusion, and behavioral data.

GAT is an open-source Matlab (The MathWorks Inc., Natick) package that provides a GUI framework to facilitate the investigation of organization of brain networks. The GUI allows users to interact with the toolbox easily without requiring knowledge of Matlab or programming. GAT provides an interactive platform for conducting graph theoretical analysis on various types of data including morphometry, functional, diffusion weighted and behavioral data (Fig. 1). The main focus of the toolbox is on analyzing the between-group differences in brain network topology. The toolbox facilitates investigation of brain networks by constructing binary undirected graphs (i.e. two regions in brain network are either connected or not connected and the connection does not have any weight or direction). However, we are extending the toolbox to be able to analyze weighted and directed networks. In the following sections, we describe the detailed procedure for analyzing structural and functional network. The procedure for analyzing between-group differences in behavioral networks/white matter networks is quite similar to the procedure described for comparison of structural morphometry/functional networks.

### Structural Graphs Based on Morphometry Data

**Figure 2:**
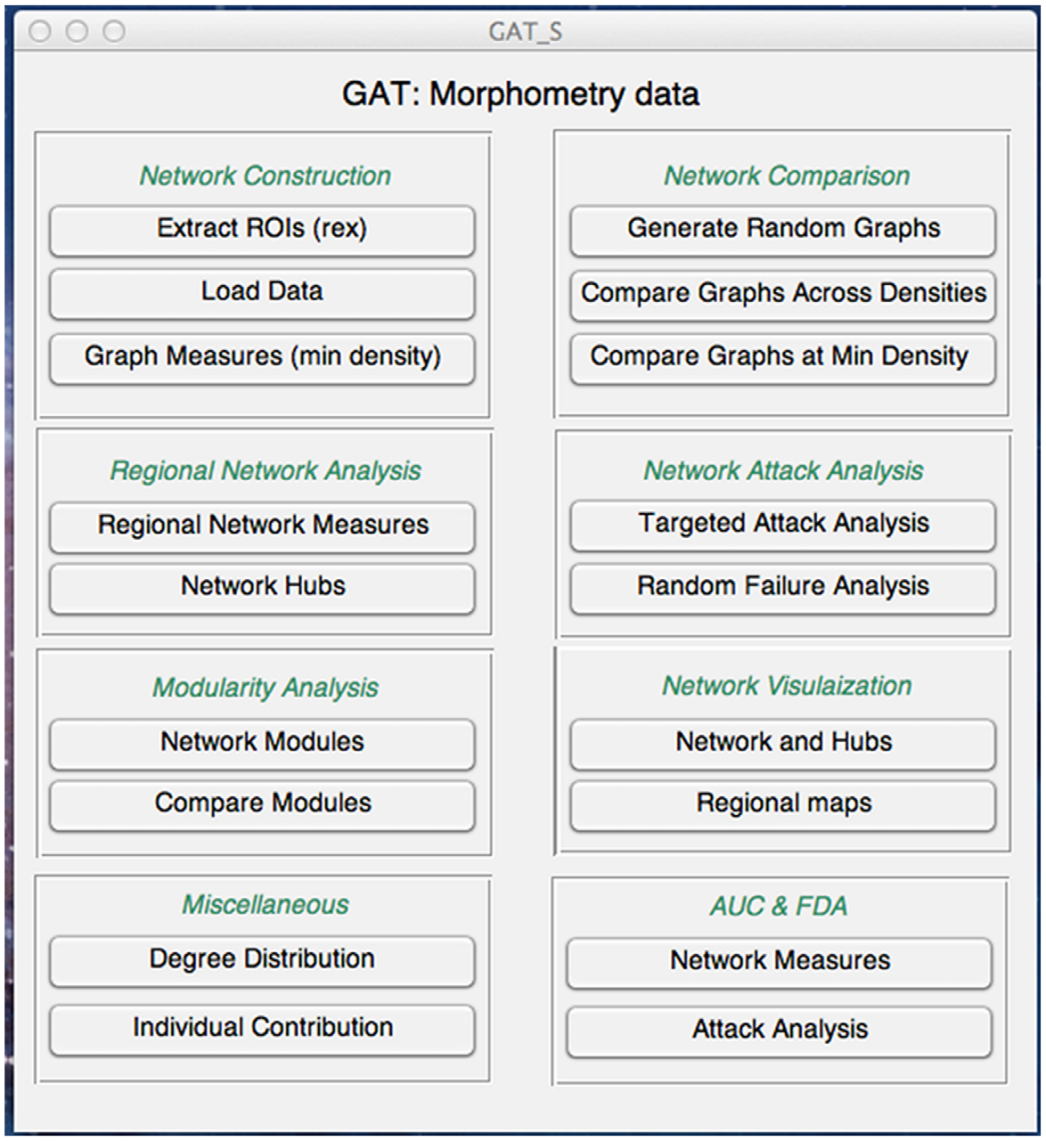
GUI for graph theoretical analysis of morphometry data. The toolbox facilitates construction of structural correlation networks, extraction of network measures, statistical analysis, regional network analysis, network failure and attack analysis, modularity analysis, AUC and FDA analyses and visualization.

Coordinated variations in brain morphology have been proposed as a valid measure to infer large-scale structural brain networks [13], [44]–[46]. This approach depends upon the assumption that positive correlations between morphometric parameters of different brain regions indicate connectivity [45]. Previous study revealed that some of the tractography maps obtained from diffusion tensor imaging are strikingly similar to pattern of correlations in cortical thickness [44]. In addition, the structural networks constructed from morphometric correlations of cortical volume, thickness, and surface data [13], [44]–[46] have been shown to follow small-world characteristics in healthy individuals [12], [17], [19], [47]. The GUI panel for investigating structural correlation networks based on morphometry data is shown in Fig. 2.

#### Input data

GAT accepts two types of morphometry data to investigate the organization of structural correlation networks: first, GAT accepts the gray matter images of individuals as input (e.g. modulated, normalized gray matter maps from voxel-based morphometry analysis). Having the gray matter images, the first step is to define the network nodes that are identified by the regional parcellation scheme that will be chosen. There are different nodal definition methods in brain network analysis. While the results might be affected by the choice of parcellation scheme, recent evidence indicates that the results of between-group comparison remain intact regardless of the applied parcellation scheme [48]. The ROI scheme that is available in GAT is the 90 cortical and subcortical regions of interest (ROIs) from the Automated Anatomical Labeling (AAL) atlas, extracted using the WFU PickAtlas Toolbox [49]. The ROIs are identical to those used in a previous graph analysis study of typical brain development by Fan and colleagues [19], [22], [50]. Note that the gray matter images should be resliced to the same dimension as that of the provided AAL ROIs. The ROIs are subsequently used to mask the individual modulated, normalized gray matter images and extract the average volume within each ROI using the REX code (http://web.mit.edu/swg/software.htm). Alternatively, a customized ROI scheme can be implemented to extract the regional volume data. REX accepts predefined ROIs as NIFTI-1 image mask files (*.img or.nii formats) or text files (.tal format). In addition, user can input previously extracted regional morphometry data (e.g. surface area, cortical thickness, regional gray matter volume data) via spreadsheets (Microsoft Excel.xls files) into GAT.

#### Covariates of nuisance

For between-group comparisons, the regional morphometry data might require correction for between-group differences in covariates of nuisance such as total brain volume, age and gender. GAT accepts the covariates of nuisance in a spreadsheet (Microsoft Excel.xls files ) and performs a linear regression analysis at every ROI to remove the effects of covariates. The residuals of this regression are then substituted for raw regional morphometry values [13], [19], [20].

#### Construction of structural correlation network

GAT uses the extracted regional morphometry data (or residuals) of all the *N* ROIs for construction of structural correlation networks. For each group, an *N*×*N* association matrix *R* is generated with each entry *r_ij_* defined as the Pearson correlation coefficient (or partial correlation coefficient) between regional values of regions *i* and *j*, across subjects. The user can specify what type of correlation analysis (Pearson vs. partial correlation) to be done on the data. From each association matrix, a binary adjacency matrix *A* is derived where *a_ij_* is considered 1 if *r_ij_* is greater than a specific threshold and zero otherwise. The diagonal elements of the constructed association matrix are set to zero. The resultant adjacency matrix represents a binary undirected graph *G* in which regions *i* and *j* are connected if *g_ij_* is unity. Therefore, a graph is constructed with *n* nodes (ROIs), with a network degree of *E* equal to number of edges (links), and a network density (cost) of D = E/[(N×(N−1) )/2] representing the fraction of present connections to all possible connections.

#### Thresholding

Thresholding the association matrices of different groups at an absolute threshold results in networks with a different number of nodes (and degrees) that might influence the network measures and reduce interpretation of between group results [29]. Two approaches are implemented for thresholding the constructed association matrices based on previous studies [12], [14], [20]: 1) Thresholding the constructed association matrices at a minimum network density (D_min_) in which all nodes become fully connected in the brain networks of both groups (none of the networks are fragmented); 2) Thresholding the constructed association matrices at a range of network densities for comparing the network topologies across that range. The GAT allows the user to specify the range and the interval between densities. The specified range may include the density points prior to the network fragmentation point but the results should be interpreted carefully.

#### Network analysis

The small-worldness of a complex network, as described in the introduction, has two key metrics: the clustering coefficient *C* and the characteristic path length *L* of the network. The clustering coefficient of a node is a measure of the number of edges that exist between its nearest neighbors. The clustering coefficient of a network is thus the average of clustering coefficients across nodes and is a measure of network segregation. The characteristic path length of a network is the average shortest path length between all pairs of nodes in the network and is the most commonly used measure of network integration [27]. To evaluate the topology of the brain network, these parameters must be compared to the corresponding mean values of a benchmark random graph [51], [52]. Thus, the small-worldness index of a network is obtained as [C/C_rand_]/[L/L_rand_] where *C_rand_* and *L_rand_* are the mean clustering coefficient and the characteristic path length of the *m* random networks [11]. *m* is the number of null networks generated for normalization of clustering and path length and is specified by the user (default value of 20). In a small-world network, the clustering coefficient is significantly higher than that of random networks (*C/C_rand_* ratio greater than 1) while the characteristic path length is comparable to random networks (*L/L_rand_* ratio close to 1). The benchmark random networks are usually constructed using rewiring algorithms that preserves the topology of the graphs; i.e. random graphs with the same number of nodes, total edges and degree distribution as the network of interest [51], [52]. However, recent evidence suggests that correlation networks are inherently more clustered and partial correlation networks are inherently less clustered than random networks of the same size and degree [53]. Thus, alternative algorithms were proposed for constructing benchmark random networks that only annihilates intrinsic structure in the empirical data. For structural networks, two types of algorithms are implemented in the GAT for construction of benchmark null networks: 1) topology randomization that generates random networks with the same number of nodes, degree, and degree distribution as the network of interest and 2) correlation matrix randomization that generates null covariance matrices that are matched to the distributional properties of the observed covariance matrix [53]. It should be noted that the choice of null network largely affects the small-world metrics for networks thresholded at lower densities (density <0.2) [53].

GAT extracts network measures using the codes developed in the Brain Connectivity Toolbox (BCT) [27]. Several network metrics including measures of network segregation (e.g. clustering, transitivity), integration (e.g. path length, efficiency), centrality (e.g. nodal betweenness, edge betweenness), and resilience (e.g. assortativity) are quantified (see [27] for a list of network measures). These metrics are quantified at both the network and regional level. GAT generates the plots of changes in global network measures as a function of network density. It also creates the association and adjacency matrices (thresholded at D_min_) for each group network.

#### Comparing network measures between groups

In order to test the statistical significance of the between-group differences in network topology and regional network measures, a non-parametric permutation test with 1000 (user defined) repetitions is used [12], [14], [20]. In each repetition, the regional data (or residuals) of each participant are randomly reassigned to one of the two groups so that each randomized group had the same number of subjects as the original groups. Then, an association matrix is obtained for each randomized group. The binary adjacency matrices are then estimated by applying the same thresholding procedure as described above. The network measures are then calculated for all the networks at each density. The differences in network measures between randomized groups (at each network density) are then calculated resulting in a permutation distribution of difference under the null hypothesis. The actual between-group difference in network measures is then placed in the corresponding permutation distribution and a one/two-tailed p-value (user defined) is calculated based on its percentile position [20]. For global network measures, GAT generates the plots of between-differences in network measures along with the quantified confidence intervals as a function of network density.

GAT also compares the areas under a curve (AUC) for each network measure [20], [54]. For this purpose, the curves extracted from thresholding across a range of densities are used. Each of these curves depicts the changes in a specific network measure (for each group) as a function of network density. To test the significance of the between-group differences in AUC of each network measure, the actual between-group difference in AUC for each network measure is placed in the corresponding permutation distribution and p-value is calculated based on its percentile position. By performing AUC analysis, the comparison between network measures is less sensitive to the thresholding process. However, the result still depends on the selected minimum and maximum network densities. While the GAT uses user-defined range of network densities for AUC analysis, we strongly recommend using the calculated D_min_ as the minimum density; specifically because the networks become fragmented below D_min_ and the comparisons might be affected by group differences in the number of nodes and connections [29]. A maximum network density of below 50% is suggested since structural networks with more than 50% connections are likely non-biological [55].

Although AUC analysis alleviates the sensitivity of the between group comparison to the thresholding process, it is too sensitive to the random structure present at higher network density values and is further insensitive to differences in the shape of the curves rather their mean. Thus, in addition to AUC analysis, GAT utilizes functional data analysis (FDA) which is a statistical method for comparing curves and overcomes these limitations [30], [31]. In FDA, each network measure curve is treated as a function (*y = f(x)*) and the summation of differences in *y*-values (a graph metric) between groups are calculated at a range of density. A nonparametric permutation test is then applied (as for AUC analysis) to test the significance of the observed summation.

The same permutation procedure is used to test the significance of the between-group differences in regional network measures. However, for regional measures, GAT performs three separate comparisons: 1) Comparing regional networks measures for the networks thresholded at D_min_; 2) comparing the AUC of the regional network measures over the specified density range; 3) performing FDA on the regional network curves over the specified density range. GAT outputs both uncorrected and false discovery rate (FDR) corrected p-values as measures of significance of the regional measures comparisons. GAT also generates the plots of between-group differences in regional network measures along with the quantified confidence intervals as a function of network density.

#### Network hub analysis

Hubs are crucial components for efficient communication in a network. Hubs are not only considered as important regulators of information flow but also play a key role in network resilience to insult [27]. Previous studies demonstrated that the hubs in the human brain structural correlation networks tend to be the regions in highly connected association cortex [13] and that the distribution of hubs are altered in structural networks of patients with Schizophrenia, Alzheimer’s disease and multiple sclerosis [12], [14], [15]. A node is considered as a hub if its regional degree/betweenness centrality is 1 (or 2) SD higher than the mean network degree/betweenness [12], [20]. GAT quantifies the network hubs based on measures of degree, betweenness centrality, local efficiency or local clustering and a user-defined SD cutoff.

The same as for regional measures, three approaches are taken for quantification of hubs. 1) The hubs are quantified for networks thresholded at D_min_; 2) the hubs are quantified based on the AUC of the degree (or betweenness, etc.) in the specified density range; 3) the hubs are quantified from FDA of the degree (or betweenness, etc.) curves in the specified density range.

#### Random failure and targeted attack analysis

To assess the resilience of brain networks to acute and focal damage, networks can be lesioned by random deletion of nodes, or by targeted attack on the highest-degree nodes in the network [56]. In GAT, random failure of the networks is simulated by randomly removing one node from the network and then measuring changes in global network metrics (e.g. size, path length, etc.) of the remaining largest component. This process is repeated by incrementally removing additional nodes randomly until the size of the largest component is 1 [3], [57].

To assess the network behavior against targeted attack, the same procedure is applied. However, in this case, the nodes are removed in rank order of decreasing nodal measure (e.g. nodal betweenness, degree, etc.). Several global metrics like size, path length, mean local and global efficiency as well as a number of nodal metrics like nodal degree and betweenness have been suggested in the literature for assessing random failure and targeted attack analysis [3], [11], [14], [20], [58]. In GAT, user may pick among several regional (e.g. degree, betweenness, clustering) and global metrics (e.g. size, mean local and global efficiency, path length) to investigate the effect of random failure and targeted attack on network behavior. The user can specify the density threshold at which random failure and targeted attack analysis are performed.

To test whether two group networks behave differently against random failure and targeted attack, a permutation analysis (same as the procedure mentioned for analyzing between-group differences in network measures) is performed. Then, the difference in network behavior is measured either at each attack (failure), through FDA or AUC analysis.

#### Network modularity analysis

Modularity is a more sophisticated measure of network segregation and is quantified by subdividing the network into groups of regions that have maximal within group connections and minimal between-group links. A unique advantage of modular organization is that it can evolve one module at a time, without risking loss of function in other modules [59]. Optimization algorithms are usually used to find such modular structures within a network. GAT uses the algorithms described in [60] and [61], and implemented in BCT, for quantification of modular structure. In order to characterize the degeneracy of the modularity structure adequately, the optimization algorithm runs several times (the number of iterations (default is 100) is specified by the user). Then, the community structure with highest maximized modularity value is used as the representative modular structure.

#### Network visualization

For visualization of networks on brain templates, GAT takes two approaches: first, GAT directly maps the network structure on an axial view of anatomical brain template (T1 image). Each ROI is represented by one node whose world coordinate has been extracted from AAL atlas. The brain networks are then mapped using these nodal dimensions. Second, GAT creates the node and edge files required by BrainNet Viewer (http://www.nitrc.org/projects/bnv/) in order to visualize the connectivity patterns. This way, you can use the files generated by GAT in BrainNet Viewer and visualize the networks and hub structures.

#### Degree distribution

Pattern of distribution of nodal connectivity in a network (degree distribution) reveals specific characteristics of the network and its resilience to random failure and targeted attacks [3], [13], [62]. Previous studies have demonstrated that the degree distribution of small-world structural brain networks follows an exponentially truncated power-law distribution [13], [63]. Such distribution is formulated as *P(d) ∼* [d^(e−1)^ * exp(-d/d_c_)], where *P(d)* is the probability of network regional degree (*d*), *d_c_* is the cut-off degree above which there is an exponential decay in probability of hubs and *e* is the exponent, and indicates a scaling regimen, followed by an exponential decay in the probability of nodes with nodal degree greater than a cutoff value of *d_c_*; suggesting a network which mostly comprised of nodes with a nodal degree close to average network degree and also a number of nodes with higher number of connections (hubs). GAT examines whether the cumulative degree distribution of the constructed networks follows an exponentially truncated power-law distribution and returns the *R*-square, which is a measure of how successful the fit is (R-square value close to 1 represents a perfect fit). It also generates a figure showing the original and fitted degree distributions. The cumulative degree distribution is used to reduce the effects of noise on smaller data sets [64]. There are also other forms of degree distribution observed in real-world networks including power law degree distribution (*d^−e^*) and exponential degree distribution (e^−e*d^). GAT also compares the R-square values for exponentially truncated power law fit model, power law fit model and exponential fit model.

### Functional Graphs Based on Resting-state or Task-based fMRI Data

**Figure 3:**
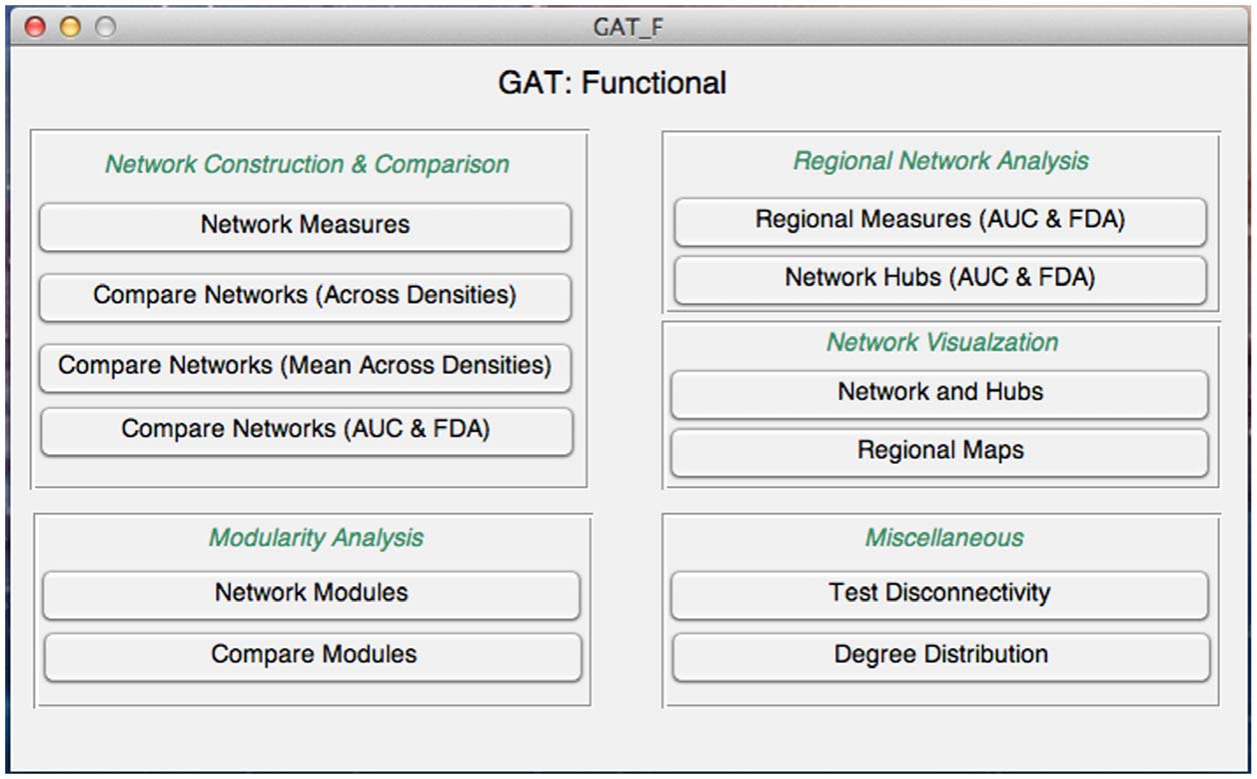
GUI for graph theoretical analysis of functional data. The toolbox facilitates construction of functional networks, extraction of network measures, statistical analysis, regional network analysis, modularity analysis, AUC and FDA analyses and visualization.

Temporal correlations in spontaneous low-frequency fluctuations in blood oxygen level dependent (BOLD) signal while subjects rest has been demonstrated as a reliable measure of brain connectivity [65]–[67]. The small-worldness of resting-state functional networks have been consistently demonstrated in several fMRI studies [3], [5], [16], [68], [69]. In addition, resting state fMRI connectivity studies are sensitive to abnormal global network organization, revealing changes in several clinical populations with cognitive deficits including Alzheimer’s disease [5], [9], schizophrenia [70], [71], traumatic brain injury [72], temporal lobe epilepsy [73], depression [10], [26] and attention-deficit hyperactivity disorder [74]. On the other hand, task-based functional connectivity pattern, i.e. temporal correlations of low-frequency BOLD signal while a subject performs a task, has also been shown to reflect the pathologic conditions. Previous studies demonstrated alterations in topologies of task-based functional connectivity pattern during memory encoding and recognition in older adults [75] and during episodic memory performance in Schizophrenia [76]. The GUI panel for investigating functional brain networks based on functional connectivity data is shown in Fig. 3.

#### Input data

GAT accepts two types of input data to investigate the organization of functional correlation networks: First, GAT accepts the output from functional connectivity toolbox (http://www.nitrc.org/projects/conn) as input and extract the individual correlation matrices (an *N*×*N* matrix) as well as the ROI names. The correlation outputs of functional connectivity toolbox are in the form of z-scores. Thus, the program first converts the z-scores to original correlation values. Network nodes are defined based on the same ROI scheme used for functional connectivity analysis within the functional connectivity toolbox. Alternatively, the user can input previously extracted correlation matrices, partial correlation values, coherence matrices, or mutual information matrices by putting them into an *M*×*N*×*N* Matlab array with *M* subjects and *N* ROIS.

#### Construction of functional network

From the previous step, we have an *N*×*N* association matrix *R* for each individual with each entry *r_ij_* represents the strength of functional connectivity between regions *i* and *j*. For each association matrix (individual), an *N*×*N* binary adjacency matrix *A* is derived where *a_ij_* is considered 1 if *r_ij_* is greater than a specific threshold and zero otherwise. The diagonal elements of the constructed association matrix are set to zero. The resultant adjacency matrix represents a binary undirected graph *G* in which regions *i* and *j* are connected if *g_ij_* is unity. Therefore, a graph is constructed with *n* nodes (ROIs), with a network degree of *E* equal to number of edges (links), and a network density (cost) of D = E/[(N×(N−1) )/2] representing the fraction of present connections to all possible connections.

#### Thresholding

Thresholding the association matrices of different individuals at an absolute threshold results in networks with a different number of nodes (and degrees) that might influence the network measures and reduce interpretation of between group results [29]. Thus, the individual association matrices are thresholded at a range of network densities.

#### Network analysis

The network analysis step is similar to analysis of structural networks. The only difference is that for functional networks, a set of network measures are quantified for each individual network, rather than each group. Therefore, normalized clustering coefficient (C/C_rand_), normalized path length (L/L_rand_), and small world index are quantified for each individual network. Benchmark random networks generated for functional graphs include topology randomization and correlation matrix randomization [53].

Similarly, the regional network measures such as nodal clustering, transitivity, path length, local efficiency, nodal betweenness and assortativity are quantified for each individual network, as described above.

#### Covariates of nuisance

For between-group comparisons, the individual network measures might require correction for between-group differences in covariates of nuisance such as age and gender. GAT accepts the covariates of nuisance in a spreadsheet (Microsoft Excel.xls files) and performs a linear regression analysis at every individual network measure to remove the effects of covariates. The residuals of this regression are then substituted for the raw individual network measures for further between-group comparison.

#### Comparing network measures between groups

Before performing statistical testing, the density range in which network comparison is meaningful needs to be identified, i.e. the density range in which the networks are not fragmented. Therefore, the individual networks are tested for fragmentation to find the range of densities in which all the nodes are connected at least to one other node. After examining all the networks, the minimum network density at which no individual network is fragmented is identified. The maximum network density is indicated by the user by examining the small-worldness of the individual networks. All the further analyses are performed on the networks thresholded over this density range.

In order to test the statistical significance of the between-group differences in network topology and regional network measures, GAT performs a parametric t-test (e.g. 2-sample t-test for comparing two groups) as well as a non-parametric permutation test with 2000 (user defined) repetitions [70]. For permutation testing, in each repetition, the network measures (or residuals) of each individual are randomly reassigned to one of the two groups so that each randomized group had the same number of subjects as the original groups. Then, the differences in network measures between randomized groups (at each network density) are then calculated resulting in a permutation distribution of difference under the null hypothesis. The actual between-group difference in network measures is then placed in the corresponding permutation distribution and a two-tailed p-value is calculated based on its percentile position. This results in a p-value of difference at each network density.

In addition to comparing global network measures at every density, FDA and AUC analyses are performed to make the between-group comparison less sensitive to the thresholding process. The FDA and AUC analyses are performed for each network measure at the specified density range. To test the significance of the between-group differences, either a nonparametric permutation test or a parametric t-test is performed.

The same procedure is used to compare the between-group differences in regional network measures. However, for the regional measures, comparison at every network density will result in a large number of comparisons (number of densities × number of ROIs). Therefore, the comparison is made on the AUC of regional network measures over the specified density range or on the FDA results. GAT outputs both uncorrected and false discovery rate (FDR) corrected p-values as measures of significance of regional measures comparisons. In the present study, the p-values reported for regional differences between groups are FDR corrected for multiple comparisons (90 comparisons).

#### Network hub analysis

The same as for structural networks, network hubs for functional networks are quantified based on measures of degree, betweenness, local efficiency or local clustering and a user-defined SD cutoff. For functional networks, the AUC of the nodal measures over the specified density range or the FDA results are used for hub analysis. Alternatively, a user-specified density can be used for hub analysis.

Other procedures such as visualization and analysis of degree distribution are similar to the one described for structural networks.

## Results

To demonstrate the capabilities of GAT, we examined the differences in organization of structural brain networks in survivors of ALL, the most common child cancer, and healthy matched controls (CON). The detailed procedures of participants, data acquisition and preprocessing is published elsewhere [34]. In summary, 28 children and adolescents with a history of ALL (age 5.0–19.8 years old) who had completed all anti-cancer treatments for at least 6 months as well as 31 healthy controls (age 4.1–18.4 years old), matched for age, gender, maternal education level and minority status, were recruited. The study was approved by the Stanford University Institutional Review Board and the Stanford Cancer Center’s Scientific Review Board and written informed consent was obtained from adult participants or from the parent/legal guardian of minor participants and assent was obtained from participants age 8 years and older per Stanford University’s regulations.

High resolution, 3D spoiled gradient recall MR images were obtained using a 3 Tesla GE Signa whole body scanner (GE Medical Systems, Milwaukee, WI) with the following parameters: repetition time = 6.436 ms, echo time = 2.064 ms, flip angle = 15°, number of excitation = 3, matrix size = 256×256 voxels, field of view = 220, slice thickness = 1.5 mm, 124 contiguous slices. To extract individual gray matter volumes, voxel-based morphometry analysis was conducted in Statistical Parametric Mapping (SPM8) [77] using the VBM8 toolbox (http://dbm.neuro.uni-jena.de/vbm). We utilized the optimized VBM process [78] which included 1) segmentation and extraction of the brain in native space, 2) normalization of the images to a standard space using a customized pediatric template, created via Template-O-Matic software [79] using images from all subjects, 3) segmentation and extraction of the normalized brain (extraction is repeated to ensure that no non-brain tissues remain), 4) modulation of the normalized images to correct for tissue volume differences due to the normalization procedure, and 5) inspection of the resulting gray matter images by expert raters, blinded to group assignment for quality, guided by boxplots and covariance matrices output by the VBM8 toolbox. The extracted gray matter volume maps were used as the input to GAT for construction and analysis of structural correlation networks.

### Network Construction

**Figure 4:**
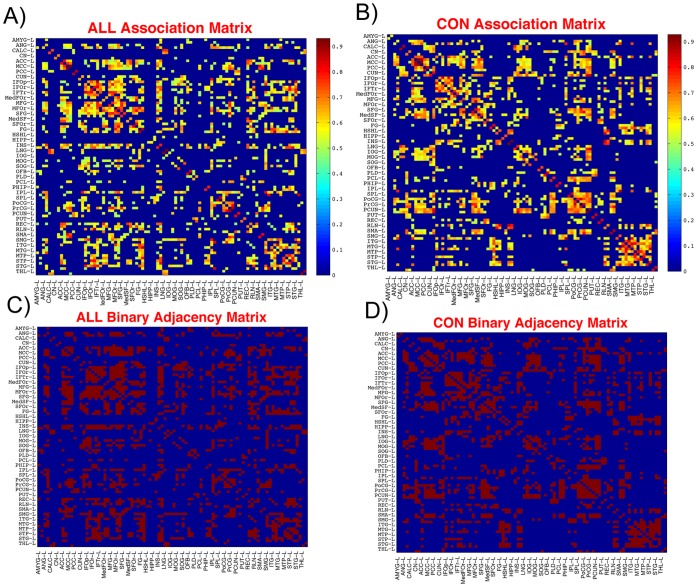
Association and adjacency matrices. Association matrices for A) ALL and B) CON groups; the color-bar shows the strength of the connections. Binary adjacency matrices for C) ALL and D) CON groups; red color represents presence of connection. These matrices are the maps thresholded at D_min_ (22%) in which all nodes became fully connected in the structural networks of both groups. For clarity, only the regions in the left hemisphere are labeled. Abbreviations are used as follow: L: left hemisphere; AMYG: amygdala; ANG: angular gyrus; CALC: calcarine fissure; CN: caudate nucleus; ACC: anterior cingulate; MCC: mid-cingulate; PCC: posterior cingulate; CUN: cuneus; IFOp: inferior frontal gyrus, opercular part; IFOr: inferior frontal gyrus, orbirtal part; IFTr: inferior frontal gyrus, triangular part; MedFOr: medial fronal gyrus, orbital part; MFG: middle frontal gyrus; MFOr: middle frontal gyrus, orbital part; SFG: superior frontal gyrus; MedSF: superior frontal gyrus, medial part; SFOr: superior frontal gyrus, orbital part; FG: fusiform gyrus; HSHL: heschl gyrus; HIPP: hippocampus; INS: insula; LNG: lingual gyrus; IOG: inferior occipital gyrus; MOG: middle occipital gyrus; SOG: superior occipital gyrus; OFB: olfactory cortex; PLD: lenticular nucleus, pallidum; PCL: paracentral lobule; PHIP: parahippocampal gyrus; IPL: inferior parietal lobule; SPL: superior parietal lobule; PoCG: postcentral gyrus; PrCG: precentral gyrus; PCUN: precuneus; PUT: putamen; REC: gyrus rectus; RLN: rolandic operculum; SMA: supplementary motor area; SMG: supramarginal gyrus; ITG: inferior temporal gyrus; MTG: middle temporal gyrus; MTP: middle temporal pole; STP: superior temporal pole; STG: superior temporal gyrus; THL: thalamus.

Regional gray matter volumes of 90 cortical and subcortical ROIs were extracted by applying AAL parcellation scheme to the individual normalized gray matter images. The extracted regional gray matter volume data were corrected for between group differences in age, gender, total brain volume, and their interactions. For each group, a 90×90 association matrix was generated by performing Pearson correlation coefficient between regional gray matter volumes across subjects. Previous studies used Pearson correlation analysis to infer statistical associations in morphometry data between brain regions [13], [15], [17]–[20], [22]. An alternative measure is partial correlation that attempts to remove the effect of indirect paths [80]. However, partial correlation is not suitable for studies with sample size smaller than the number of ROIs [53]. Binary adjacency matrices were then derived by thresholding the association matrices at a range of densities (D_min_: 0.01∶0.45; D_min_ = 0.22). The lower bound of the range is determined as the minimum density in which the networks of both groups are not fragmented (D_min_ = 0.22). For densities above 0.45 the graphs becomes increasingly random (small-world index <1.5). Additionally, for anatomical networks, connections above this density are less likely biological [81]. Each of the derived binary adjacency matrices represents a network with a specific density. The resultant association and binary adjacency matrices (thresholded at D_min_) are shown in Fig. 4. Consistent with previous studies [12]–[14], [17], [20], the association matrices in both groups exhibited similar patterns of connectivity, with generally strong correlations between bilaterally homologous regions as well as between regions within the same lobe.

### Within-group Global Network Measures

**Figure 5:**
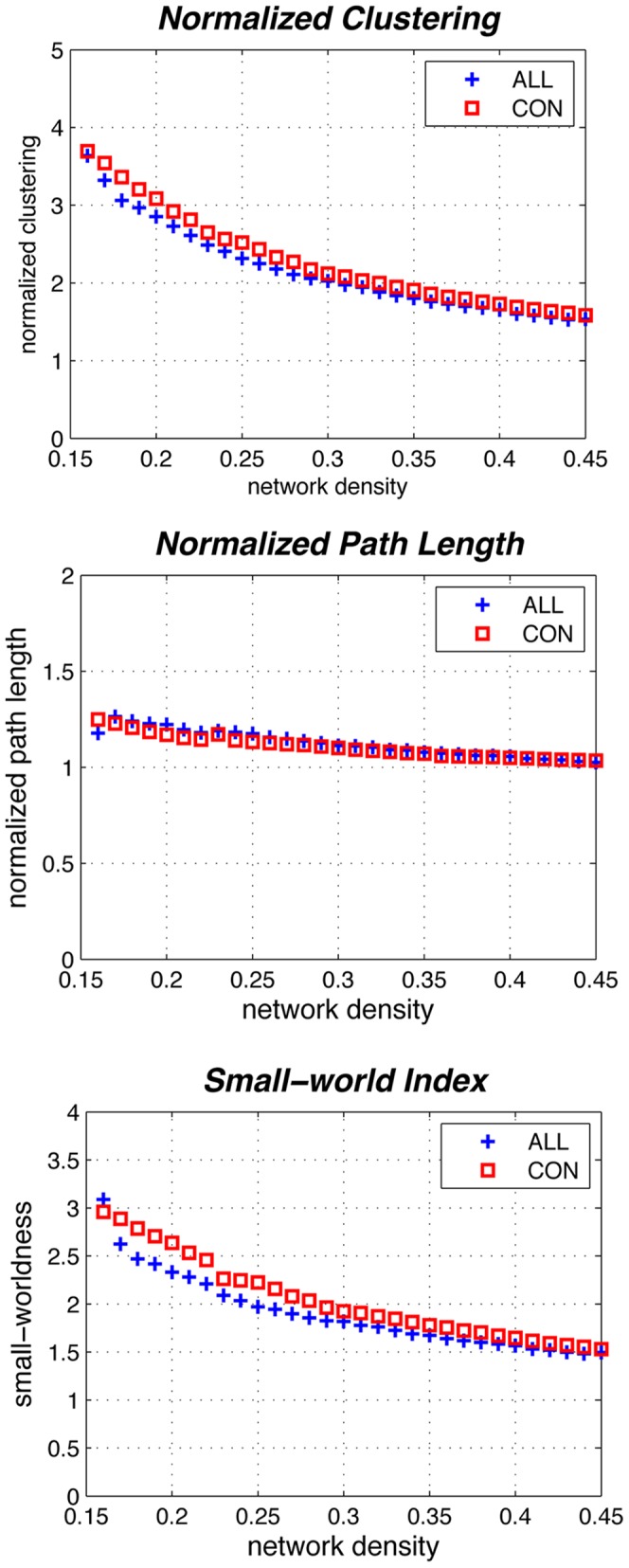
Changes in global network measures as a function of network density. Normalized clustering (top), normalized path length (middle), and small-world index (bottom) of both the ALL and CON networks. Both the networks follow a small-world organization, i.e. normalized clustering of greater than 1 and normalized path length of close to 1.

The minimum network density below which the networks are fragmented was D_min_ = 0.22. To investigate changes in the network topology as a function of network density, GAT thresholds the constructed association matrices at a range of network densities. Changes in global network measures as a function of network cost are shown in Fig. 5. The networks of both groups followed a small-world organization across a wide range of network densities; both the networks had a path length slightly higher than random networks while having a clustering coefficient that was much higher than that in random networks. This pattern results in a small-world index of higher than one across the range of network densities.

### Between-group Differences in Global Network Measures

#### Differences across network densities

**Figure 6:**
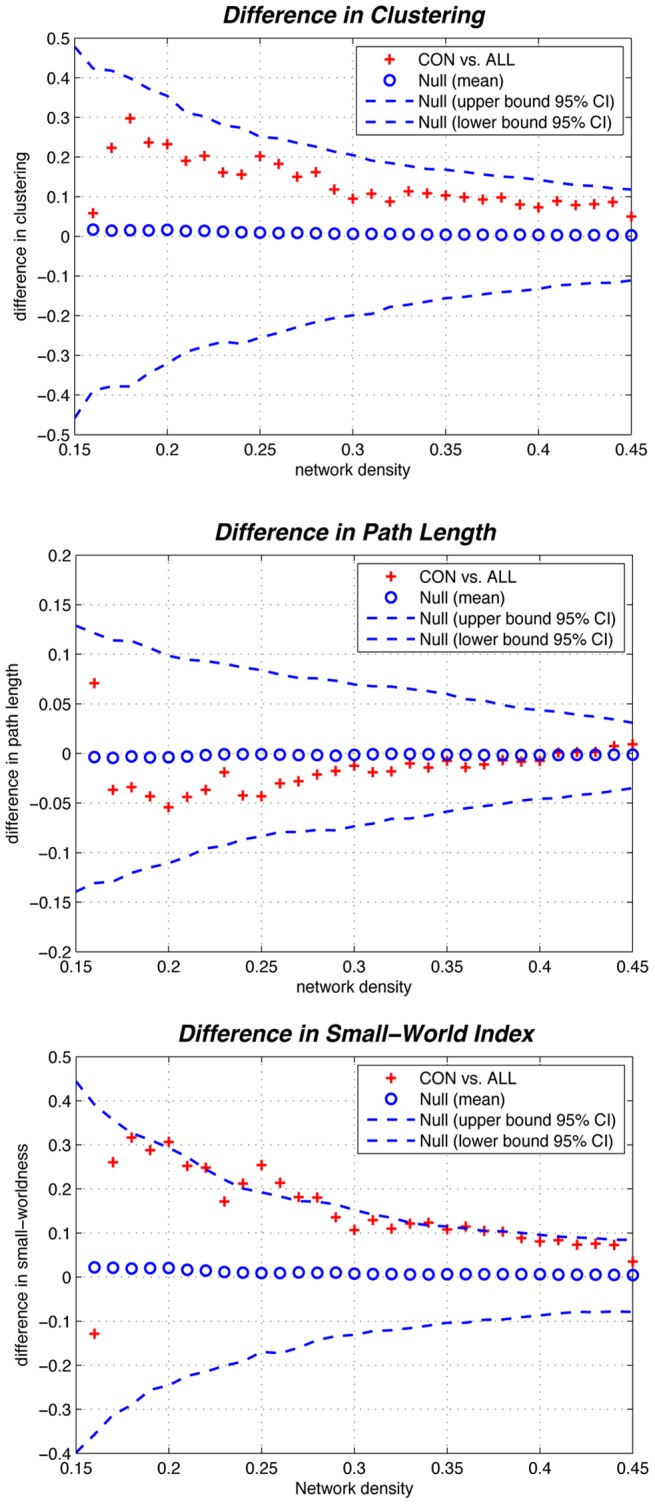
Between-group differences in global network measures as a function of network density. The 95% confidence intervals and between-group differences in normalized clustering (top), normalized path length (middle) and small-world index (bottom). The + marker shows the difference between CON vs. ALL networks; the + signs falling out of the confidence intervals indicate the densities in which the difference is significant. The positive values indicate CON > ALL and negative values indicate CON < ALL. Relative to CON network, the small-world index in ALL network was significantly lower in various network densities. The AUC of the small-world index was also significantly lower in the ALL network.

We investigated between-group differences in global network measures on networks thresholded at a range of densities (0.22∶0.01∶0.45). Compared with CON, the ALL network showed smaller normalized clustering and larger normalized path length across the range of densities but the difference was not significant (p<0.05) (Fig. 6). This pattern led to a significantly smaller small-world index in the ALL network at several densities across the range (p<0.05) (Fig. 6).

#### FDA and AUC analysis on global network measures

In addition to comparing networks at various densities, we compared the AUC for global network measure curves (density range of 0.22∶0.01∶0.45) between groups. Similar to the observed differences across densities, while the ALL network had non-significantly smaller AUC for normalized clustering (p = 0.079) and larger normalized path length (p = 0.352), it showed a significantly smaller small-worldness (p = 0.043) compared with CON network. The FDA results were also consistent with the AUC results; ALL network had non-significantly smaller normalized clustering (p = 0.080), larger normalized path length (p = 0.352), and significantly smaller small-world index (p = 0.041).

### Between-group Differences in Regional Network Measures

#### Differences at D_min_

[Thightest]We investigated between-group differences in regional network measures, specifically nodal betweenness, on networks thresholded at D_min_. Regions including left middle frontal gyrus, left medial superior frontal and right supramarginal gyrus showed significantly smaller betweenness in ALL network. Conversely, a number of regions including right insula, right inferior parietal and left supramarginal gyrus showed significant larger betweenness in ALL network. None of these regions survived after correction for multiple comparisons (P<0.05).

#### FDA and AUC analysis on regional network measures

**Figure 7:**
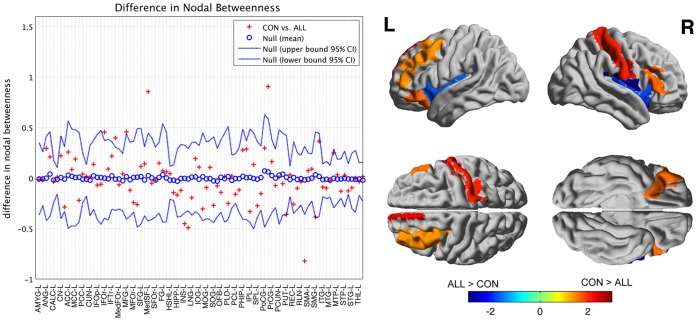
Between-group differences in regional network topology. Regions that showed significant between-group differences in nodal betweenness mapped on ICBM152 surface template. Hot color identifies the regions that have significantly higher nodal betweenness in CON compared to ALL while cold color identifies regions with significantly higher nodal betweenness in ALL compared to CON. The regional differences were quantified based on AUC analysis in the density range of 0.22∶0.01∶0.45.

We also compared the AUC for regional network measure curves (density range of 0.22∶0.01∶0.45) between groups (Fig. 7). While some of the regions were common between AUC analysis and analysis of network differences at D_min_ a number of different regions were also found. Specifically, left orbital inferior frontal, and right triangular inferior frontal regions showed significantly smaller betweenness in ALL while left insula showed significantly larger betweenness in ALL. None of these regions survived after correcting for multiple comparisons (p<0.05) so the regional results are considered exploratory. The FDA results were similar to AUC results.

### Network Hubs

We considered a node as a hub if its regional betweenness centrality is 2 SD higher than the mean network betweenness. Hubs were quantified for networks thresholded at D_min_ as well as based on FDA and the AUC of nodal betweenness curves (density range of 0.22∶0.01∶0.45). For networks thresholded at D_min_, the ALL network hubs were found in the right inferior parietal lobule, left supramarginal gyrus, left postcentral gyrus and right rolandic operculum whereas the CON network hubs were found in the right cuneus, left middle frontal gyrus, left medial superior frontal gyrus, bilateral postcentral gyrus, and right supramarginal gyrus.

**Figure 8:**
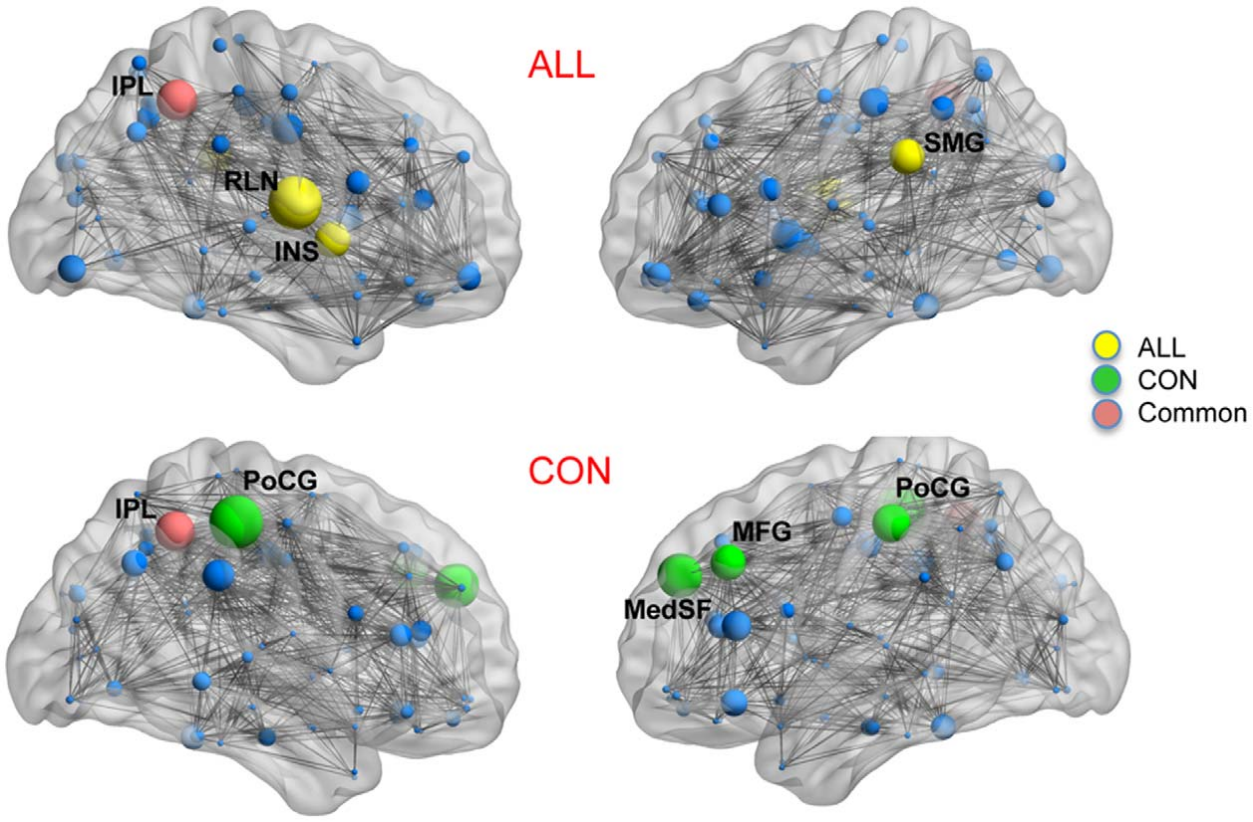
Network hubs.: Constructed networks and corresponding hubs for ALL (top) and CON (middle) groups. Nodes that are labeled represent network hubs (nodes with nodal betweenness of 2SD greater than network mean betweenness). The volume of the spheres represents the betweenness of the corresponding brain region. Green color highlights hubs specific to CON network; yellow color represents hubs specific to ALL network; Orange color represents hubs that are common in both groups; blue color represents non-hub regions. The hubs were quantified based on AUC analysis in the density range of 0.22∶0.01∶0.45.

Hub quantification based on AUC analysis revealed ALL network hubs in the right insula, right inferior parietal, right rolandic operculum and left supramarginal gyrus and CON network hubs in the left middle frontal gyrus, left medial superior frontal gryus, right inferior parietal lobule and bilateral postcentral gyrus (Fig. 8). Hubs quantified using FDA were exactly the same as those obtained from AUC analysis.

### Random Failure and Targeted Attack Analysis

**Figure 9:**
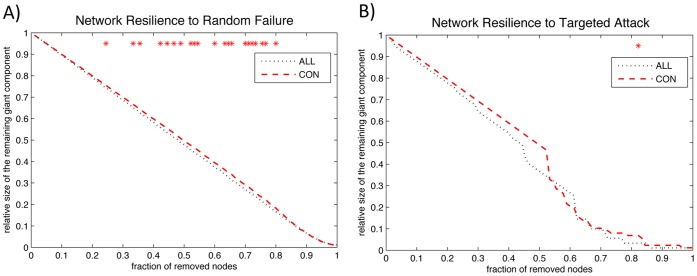
Between-group differences in network resilience to random failure and targeted attack. Changes in the size of the largest component of the networks after cascading random failure (A) and targeted attack (B). Stars show where the difference in the size of the largest remaining components between groups is significant. The ALL network shows less tolerance to random failure and targeted attack. The AUC analysis revealed a significantly lower tolerance to random failure in the ALL network compared to CON.

In order to analyze the networks behavior in response to random failure, we calculated the size of the largest remaining component in response to successive removal of nodes, in random order. Changes in size of the remained largest component of the network as a function of fraction of randomly removed nodes are depicted in Fig. 9A. In most of the fractions of removed nodes, the resilience of the ALL network to random failure was significantly reduced compared to CON (p<0.05). The AUC of the curve was also significantly lower in the ALL network compared to CON (p = 0.043). The FDA results were also consistent with the AUC results showing significantly lower robustness to random failure in the ALL network (p = 0.042).

The same procedure was applied to analyze the networks behavior in response to targeted attack but removing the nodes in rank order of decreasing nodal betweenness centrality. The CON network was generally more robust to targeted attack compared to ALL network and the difference reached significance at a few fractions of attacked nodes (p<0.05) (Fig. 9B). The AUC for the CON network was larger than for the ALL but it was not significant (p = 0.175). The same results were observed using FDA.

### Network Modularity Analysis

**Figure 10:**
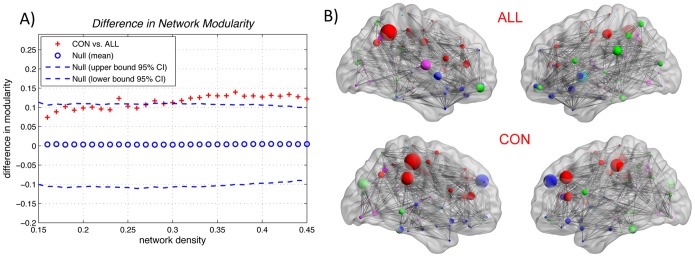
Between-group differences in network modularity. The 95% confidence intervals and between-group differences in network modularity (A). The + marker shows the difference between CON vs. ALL networks; the + signs falling out of the confidence intervals indicate the densities in which the difference in global modularity is significant. The positive values indicate CON > ALL and negative values indicate CON < ALL. The modular structures of the CON and ALL networks are also shown (B). While both the networks had the same number of modules (five modules color-coded for each network separately), the degree of modularity is significantly lower in the ALL group in various network densities relative to CON group.

Global network modularity was significantly higher in the CON network compared with the ALL across several network densities (Fig. 10A). The FDA and AUC analysis also revealed a significantly higher modularity in the CON network in the density range of interest (0.22∶0.01∶0.45) (p = 0.023 for AUC analysis and p = 0.024 for FDA). Five modules were found in the networks of both groups as color-coded in Figure 10B.

### Degree Distribution

**Figure 11:**
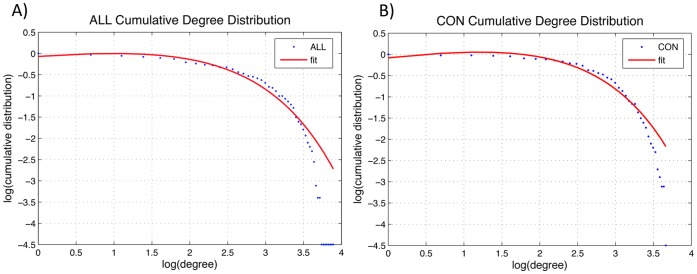
Degree distributions. The log-log plot of cumulative degree distributions of ALL (A) and CON (B) networks thresholded at D_min_. The solid line indicates the exponentially truncated power-law curve fitted to the cumulative degree distribution of the networks (dotted line). The estimated exponent was 1.19 for ALL and 1.27 for CON, the cut-off degree was 2.65 for the ALL and 2.52 for the CON network. These parameters resulted in R-square value of 0.97 for both distributions (value close to one represents a good fit).

The degree distribution in both networks followed an exponentially truncated power-law distribution (*P(d) ∼* [d^(e−1)^ * exp(−d/d_c_)]) (Fig. 11). The exponent estimate (*e*) was 1.19 for ALL and 1.27 for CON networks. The cut-off degree (*d_c_*) was 2.65 for ALL network and 2.52 for the CON. The R-square value was 0.97 for both groups’ distributions fits. We also compared the goodness-of-fit for other forms of degree distribution. The R-square for BC group was 0.56 for power law fit and 0.91 for exponential fit. For the CON group, the R-square value was 0.50 for power law fit and 0.85 for exponential fit.

## Discussion

Graph theoretical analyses of neuroimaging data have increased our understanding of the organization of structural and functional brain networks in recent years. In this report, we describe the development of a graph analysis toolbox (GAT) that facilitates analysis and comparison of structural and functional brain networks. We demonstrated the capabilities of GAT by investigating the differences in organization of structural brain networks in survivors of acute lymphoblastic leukemia (ALL) and healthy matched Controls (CON). The results revealed an alteration in small-world characteristics of the brain structural networks in the ALL network; an observation that confirms our hypothesis suggesting widespread neurobiological injury in ALL survivors. This is the first report of altered large-scale structural brain networks in ALL survivors.

### Large-scale Structural Networks in ALL

#### Global network measures

Both the ALL and CON networks followed a small-world organization across a wide range of densities (Fig. 5). Such a network allows for efficient information processing by providing an optimal balance between segregation and integration [14]. The results are in line with previous graph analysis studies that have consistently shown a small-world architecture in structural brain networks in healthy individuals [12], [19], [47].

Compared to the CON network, the ALL network showed smaller normalized clustering and larger normalized path length that led to a significant smaller small-world index across several densities in the ALL network. In addition, the area under the small-worldness curve was also significantly smaller in the ALL network suggesting the consistency of the findings irrespective of the threshold. These findings suggest that the structural networks of ALL patients tend to have a more regularized configuration relative to CON group; a configuration that is more segregated but less integrated compared to random networks. This configuration in the ALL network is thus less optimal for information processing compared to CON network. Our results corroborate previous structural neuroimaging findings that have demonstrated a diffuse pattern of atrophy in white matter and gray matter structure in ALL survivors [34]–[38].

One potential mechanism underlying this network-level alteration is white matter damage. A large body of evidence suggests that global gray matter atrophy is associated with focal and global white matter damage due in part to the transection of axons and subsequent retrograde neuronal loss [82], [83]. Animal studies show that chemotherapy suppresses neural progenitor cell proliferation responsible for white-matter tract integrity and cortico-cortical connections [84]–[88]. Thus, the observed network-level alteration in structural correlation network of ALL patients might arise from neurotoxic effects of chemotherapy on cortico-cortical connections. This idea is also supported by previous diffusion weighted imaging studies involving ALL survivors that reported a diffuse pattern of microstructural white matter damage [35], [37], [89].

#### Regional network measures

The observed between-group differences in regional network measures did not survive after correction for multiple comparisons. However, the uncorrected results were in line with previous findings and thus we consider the following to be exploratory. Several regions in the prefrontal cortex showed smaller betweenness centrality in the ALL network compared to CON. Nodes with higher centrality in the network identify regions that have the potential to participate in a large number of functional interactions [90]. A number of neuropsychological studies have shown a subtle long-term neurocognitive deficits, specifically in cognitive functions subserved by prefrontal cortex, in survivors of childhood ALL (see [41], [42] for a review). Previous neuroimaging studies reported reduced white matter connectivity between frontal and occipital regions [34], [89], decreased white matter structure and volume in frontal regions [37], [38], [91], and reduced fractional anisotropy in frontal white matter structure [37] in ALL survivors. Decreased regional white matter volume in prefrontal regions was also associated with decreased performance on neuropsychological measures in this population [91]. Paakko and colleagues [92] reported that ALL patients with white matter changes more often had impairment of attention and cognitive functions subserved by the frontal regions. These reports might explain the observed lack of centrality in prefrontal regions in the ALL network in our study suggesting an impaired functional interaction between frontal regions and the rest of the brain network.

#### Network hubs

The identified hubs in the CON network are consistent with the results of previous graph-theoretical analysis involving healthy subjects [12], [14]. The CON network showed more number of central hubs located in the prefrontal cortex compared to ALL. The observed lack of highly central hubs in the prefrontal cortex in the ALL network is in line with the results of group-differences in regional network measures and suggests network alterations involving regions critical for the executive functions in ALL.

#### Random failure and targeted attack analysis

The AUC of the random failure curve was significantly lower in the ALL network compared to CON suggesting the overall lower resilience of the ALL network in response to random failure. In addition, the resilience of the ALL network was lower in response to targeted attack but the overall between-group difference was not significant. This observation is consistent with the results of global network measures suggesting that the ALL network is more regularized relative to CON network. Networks with more regularization are less resilient to random failure and show reduced resilience to pathologies [20]. A regularized network, compared with a small-world network, does not have highly connected hubs and thus fails to integrate various modules in the event of network fragmentation as a result of cascaded random failures.

#### Network modularity

While both the networks had the same number of modules, the degree of network modularity was significantly smaller in the ALL network. The smaller network modularity in the ALL network corroborates the observed higher path length and lower clustering in the ALL network and suggests a reduction in the balance between network segregation and integration in ALL.

#### Degree distribution

The degree distribution of both networks followed an exponentially truncated power law distribution suggesting a network with many regions having small number of connections and a few regions having large number of connections (hubs). The cutoff degree was around 3 for both the networks, consistent with previous reports [13], [20].

### GAT Features

GAT provides a GUI framework to facilitate the investigation of organization of brain networks without requiring the users to have knowledge of Matlab or programming. In addition, GAT has a number of unique features that other available graph-analysis packages lack. First, unlike other graph-analysis packages that mainly focused on extraction of network measures and/or visualization, the unique feature of the GAT is that it integrates the processes of ROI extraction, network construction, regional and global network analysis, network comparison, hub analysis, random failure and targeted attack simulation and network visualization. Second, GAT facilitates analyzing between-group differences in global network measures, regional network properties, and resilience to random failure and targeted attack using permutation analysis. Third, in addition to comparing networks at various densities, GAT compares the areas under a curve (AUC) for each network measure. By performing AUC analysis, the comparison between network measures is less sensitive to the thresholding process. GAT performs AUC analysis for comparing global networks measures, regional network measures, network hubs as well as random failure and targeted attack analysis. Fourth, GAT utilizes FDA which is more sensitive to differences in shape of the curves to complement the AUC results. Finally, GAT provides tools for testing network connectivity and analyze network degree distribution.

However, GAT mainly performs network comparison by converting the association matrices to binary undirected adjacency matrices, which result in loss of information. Comparing weighted and/or directed networks would be more informative but there are still some methodological challenges to analyze and compare weighted and directed networks [93]. We are extending the toolbox to be able to analyze weighted and directed networks.

In summary, we described the development of GAT that facilitates analysis and comparison of structural and functional brain networks. Using GAT, we compared the organization of large-scale structural correlation networks between ALL survivors and matched healthy controls. We found an alteration in small-world characteristics of the structural correlation networks in ALL relative to healthy controls that corroborates our hypothesis suggesting widespread network-level neurobiological injury in ALL survivors. Compared to CON, the ALL network showed smaller clustering and longer path length that resulted in significantly smaller small-world index in ALL group; an observation that suggests a more regularized network structure in ALL relative to CON network. The ALL network is thus less optimal for information transfer and more vulnerable to network failures and attacks, supported by random failure and targeted attack analyses results.
